# Increased Levels of Caspase-1 and IL-1β Among Adults With Persistent Immune Activation After 12 Years of Suppressive Antiretroviral Therapy in the Infectious Diseases Institute HIV Treatment Cohort

**DOI:** 10.1093/ofid/ofad539

**Published:** 2023-11-07

**Authors:** Rose Nabatanzi, Phillip Ssekamatte, Barbara Castelnuovo, Andrew Kambugu, Damalie Nakanjako

**Affiliations:** Department of Immunology and Molecular Biology, Makerere University College of Health Sciences, Kampala, Uganda; Infectious Diseases Institute, Makerere University College of Health Sciences, Kampala, Uganda; Department of Immunology and Molecular Biology, Makerere University College of Health Sciences, Kampala, Uganda; Infectious Diseases Institute, Makerere University College of Health Sciences, Kampala, Uganda; Department of Medicine, School of Medicine, Makerere University College of Health Sciences, Kampala, Uganda; Infectious Diseases Institute, Makerere University College of Health Sciences, Kampala, Uganda; Department of Medicine, School of Medicine, Makerere University College of Health Sciences, Kampala, Uganda; Infectious Diseases Institute, Makerere University College of Health Sciences, Kampala, Uganda; Department of Medicine, School of Medicine, Makerere University College of Health Sciences, Kampala, Uganda

**Keywords:** caspase-1, inflammasome byproducts, persistent immune activation_1_, subclinical replication

## Abstract

**Background:**

We sought evidence of activated pyroptosis and the inflammasome pathways among human immunodeficiency virus (HIV)–infected adults after 12 years of suppressive antiretroviral therapy (ART) and persistent immune activation in the Infectious Diseases Institute HIV treatment cohort in Uganda.

**Methods:**

In a cross-sectional study, using peripheral blood mononuclear cells of HIV-infected individuals with high and low immune activation (CD4/CD8^+^CD38^+^HLA-DR^+^ cells) relative to HIV-negative reference group, caspase-1 expression was measured using flow cytometry and plasma interleukin 18 and interleukin 1β (IL-1β) levels using enzyme-linked immunosorbent assay.

**Results:**

There was higher expression of caspase-1 by CD4 T cells of ART-treated individuals with high immune activation relative to those with lower immune activation (*P* = .04). Similarly, plasma levels of IL-1β were higher among ART-treated individuals with high immune activation levels relative to those with low immune activation levels (*P* = .009). We observed a low positive correlation between caspase-1 expression by CD4/CD8 T cells and immune activation levels (*r*  *=* 0.497 and *r*  *=* 0.329, respectively).

**Conclusions:**

Caspase-1 and IL-1β were high among individuals with high immune activation despite 12 years of suppressive ART. There is a need to further understand the role of persistent abortive infection and the latent HIV reservoir characteristics as drivers of persistent activation and inflammation and to subsequently intervene to prevent the complications of chronic immune activation during long-term ART.

## Teaser

Elevated caspase-1 and IL-1β were observed among individuals with high immune activation despite 12 years of suppressive antiretroviral therapy.

**Figure 1.**
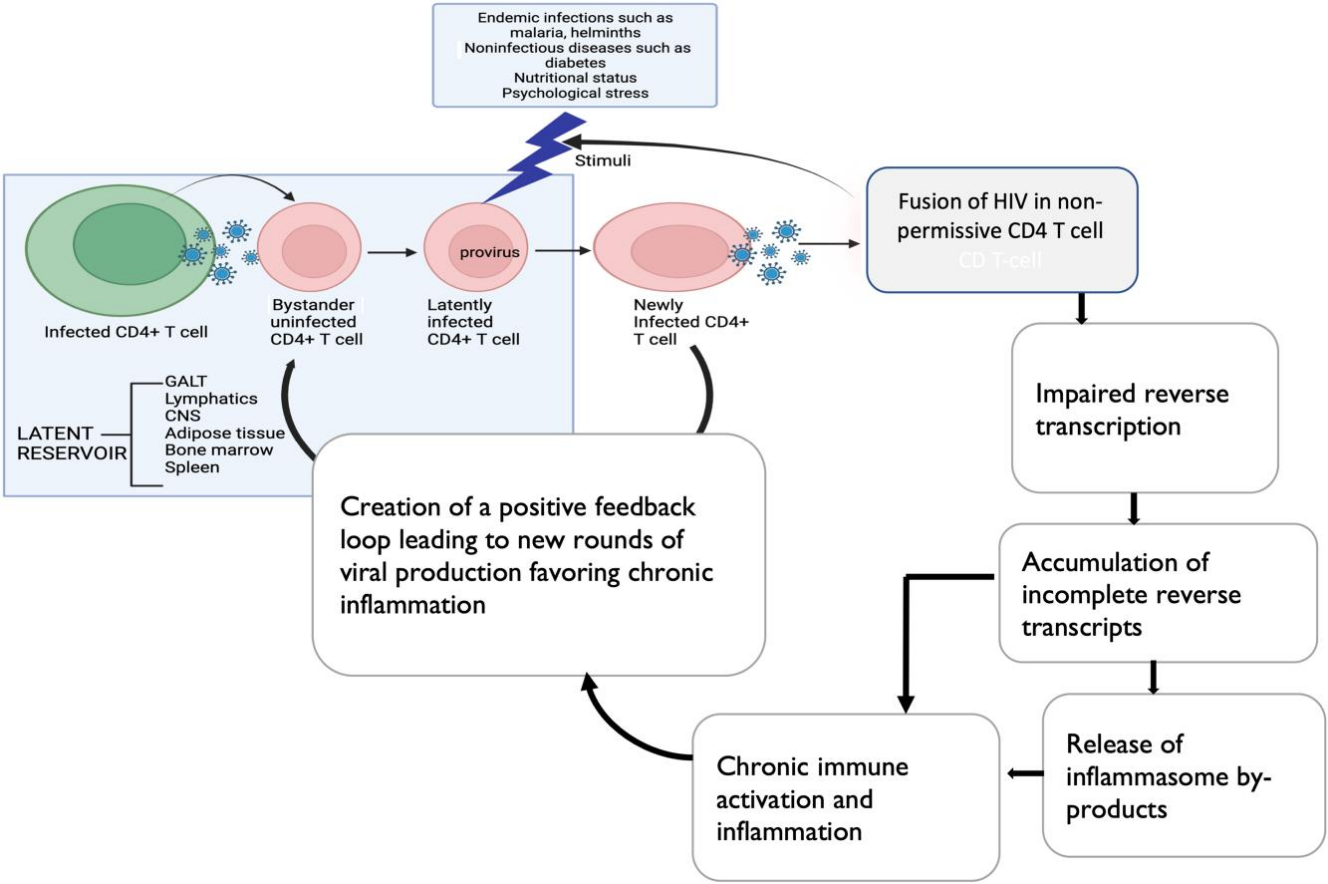
Schematic illustration of chronic immune activation and pyroptosis cell death pathway among antiretroviral therapy (ART)–treated adults after 12 years of suppressive ART in the Infectious Diseases Institute HIV treatment cohort. Presence of endemic infections in sub-Saharan Africa stimulates ongoing low levels of abortive infection and pyroptotic cell death seeded by abortive infection contributing to chronic immune activation and inflammation observed among African ART-treated adults. Abbreviations: CNS, central nervous system; GALT, gut-associated lymphoid tissues; HIV, human immunodeficiency virus.

Antiretroviral therapy (ART) has dramatically increased the life span of human immunodeficiency virus (HIV)–infected individuals worldwide [1, 2]. Similarly, AIDS-defining illnesses and opportunistic infections have greatly declined [3, 4]. Despite this improvement, defects persist in the immune system placing ART-suppressed individuals at higher risk of diseases related to immune activation and inflammation [5–7]. In our previous work, we found increased levels of the systemic immune activation markers soluble CD14 and CD69 and elevated levels of interleukin 6 (IL-6) and C-reactive protein, despite long-term suppressive ART and restored CD4 T cells to relatively normal CD4 counts of ≥500 cells/µL [8, 9]. The reasons for the persistent immune activation and inflammation are not clear in the absence of the known drivers of immune activation including circulating intestinal fatty acid–binding protein, cytomegalovirus viremia, and microbial translocation [8]. We therefore hypothesize that ongoing production of low levels of HIV virus from the pool of latently infected cells in tissues might contribute to presence of short HIV transcripts in circulation that drive persistent immune activation and inflammation [10–12], despite peripheral viral suppression (Figure 1).

Viral proteins have been shown to reduce by >99% upon ART initiation [13]; therefore, presence of viral proteins in circulation is likely due to replenishment, which may play a role in activation of the immune system [14]. Similarly, low production of virions from reactivated reservoir cells could result in abortive infection in the presence of ART, leading to activation of inflammasome and proinflammatory caspases responsible for release of proinflammatory cytokines (interleukin 1β [IL-1β] and interleukin 18 [IL-18]) [15]. Caspase-1 activation can lead to cleavage of gasdermin-D resulting in pyroptosis, a highly inflammatory form of programmed cell death [16, 17]. Pyroptotic cell death can be induced by various stimuli that activate inflammasome. The activation of NLRP3 prompts its binding to ASC and caspase-1, forming the inflammasome. Caspase-1 processes pro-IL-1β and pro-IL-18 to their active forms [18]. Although pyroptosis has been described in association with poor CD4 cell recovery [19], little is known about its contribution to chronic inflammation among ART-treated individuals in sub-Saharan Africa (SSA) where several causes of inflammation (malaria, tuberculosis, and helminths) are endemic [20]. Immune activation and inflammation are considered a positive response in the presence of an antigen but their persistence is linked to increased apoptosis, senescence, exhaustion, and anergy of immune cells in vivo [21, 22]. Pyroptosis can stimulate new rounds of pyroptosis creating a feed-forward positive loop favoring chronic inflammation [23]. Additionally, both phenotypes have been associated with fatal non-AIDS illnesses such as cardiovascular diseases, malignancies, and multiorgan damage among adults aging with HIV [24–26].

We therefore described the evidence of activated pyroptosis and the inflammasome pathway among HIV-infected adults after 12 years of suppressive ART (with viral load <50 copies/mL) and persistent immune activation in the Infectious Diseases Institute (IDI) HIV treatment cohort in Uganda. Understanding the relationship between pyroptosis and persistent immune activation and inflammation during long-term ART provides more insight into the potential drivers of the inflammasome pathway and immunological complications of persistent immune activation and inflammation among individuals aging with chronic HIV and ART [24–26].

## METHODS

### Study Setting

We analyzed frozen peripheral blood mononuclear cells (PBMCs) from ART-treated HIV-infected individuals within the IDI HIV treatment cohort located at the Mulago national referral hospital in Kampala, Uganda, who initiated ART at CD4 counts <350 cells/μL, as previously described by Kamya et al 2007 [27]. Blood was drawn from 58 ART-treated adults who had HIV viral suppression (viral load <50 copies/mL) for 12 years and had no opportunistic infections in the preceding 6 months. Ten age- and sex-matched HIV-negative persons from the same community were used to provide background immune activation levels in the HIV-negative community. HIV-negative persons were randomly selected from sex- and age-matched adults from the same community (family members and neighbors of the ART-treated adults in the IDI cohort) to provide a reference for background levels of immune activation in the community, which was denoted as “low immune activation” for this study. Immune activation levels above the levels in the HIV-negative controls were denoted “high immune activation.”

### Participants

Of the 58 ART-treated individuals analyzed, 38 (65%) were female and the median age was 49 (interquartile range [IQR], 39–65) years, with CD4 T-cell recovery ranging from 58 to 1587 cells/µL. The HIV-negative persons were age and sex matched (±5 years), with a median age of 53 (IQR, 40–62) years.

### Experimental Procedure

**Figure 2.**
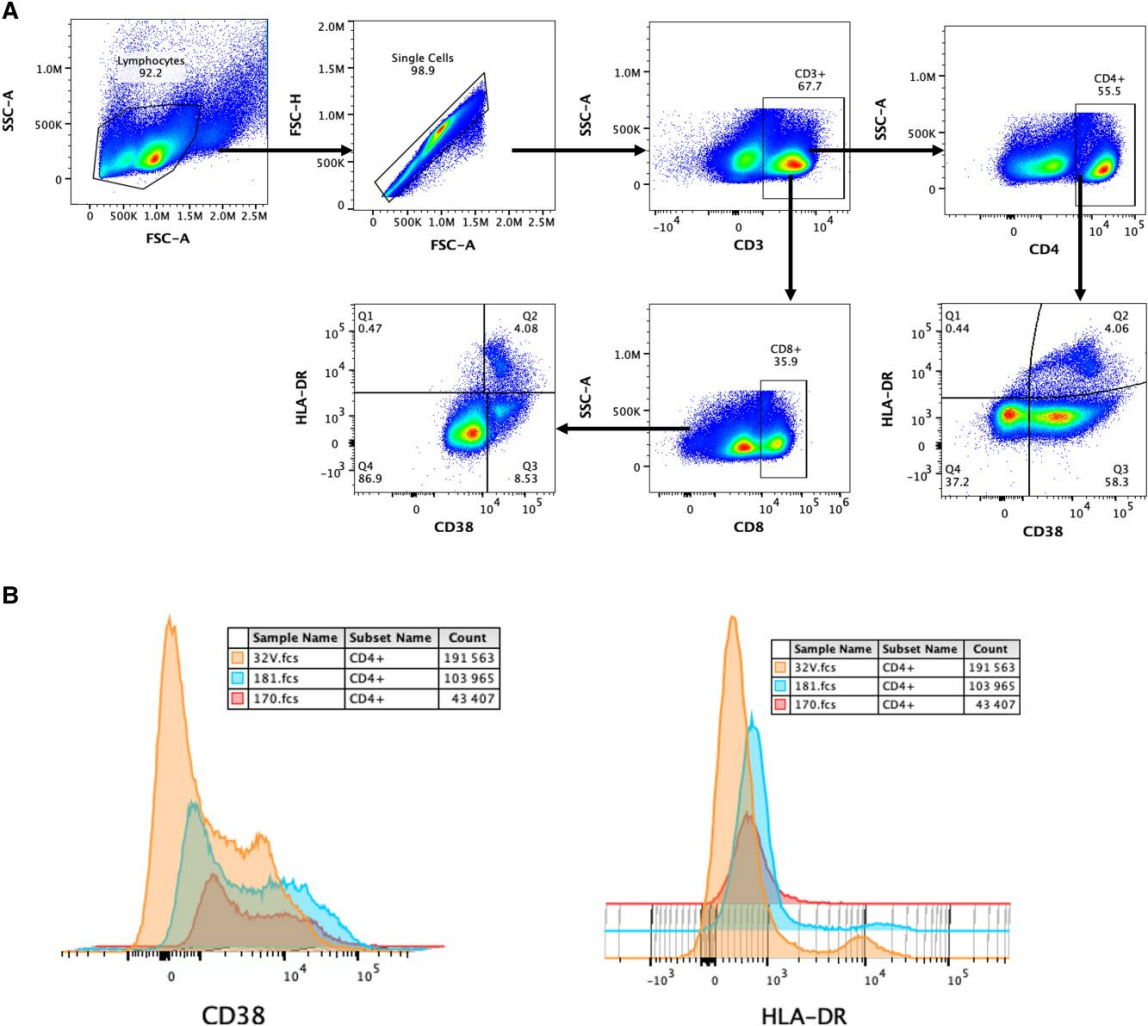
Gating strategy for immune activation phenotypes. *A*, Lymphocyte population followed by the singlet gate, CD4/CD8 T-cell gate off the CD3 T cells, and the immune activation as denoted by CD38^+^ and HLA-DR^+^cells. *B*, Comparisons of expression of CD38 and HLA-DR on diferent sample types.

To determine immune activation, cell surface staining was done after optimizing the use of zombie yellow BV570 live/dead cell viability staining kit and monoclonal antibodies: CD3, CD4, CD8, HLA-DR, and CD38 (all from Biolegend). Surface staining was done at 4°C for 30 minutes. Cells were washed with staining buffer (5% fetal bovine serum, 0.01% sodium azide, and 1× PBS). Samples were acquired on an Cytoflex LX Beckman Coulter and data analyzed using FlowJo software (Tree Star, version 10.1). Compensation controls were used to correct for spectral overlap (Figure 2).

For measurement of activated caspase-1, thawed PBMCs were incubated with FAM-YVAD-FM (Abcam), which binds irreversibly to active caspase-1. Markers of pyroptosis and the inflammasome pathways (caspase -1, IL-1β, IL-18 and IL-18Pa) were measured among ART-treated individuals with high and low immune activation. Changes in fluorescence intensity were measured by Cytoflex LX Beckman Coulter flow cytometry. Plasma levels of IL-18, IL-18BPa, and IL-1β as byproducts of inflammasome activation were measured using the Quantikine enzyme-linked immunosorbent assay kit (R&D Systems).

### Statistical Analysis

Flow cytometry data were analyzed using FlowJo software version 10.1 and exported to an Excel sheet that was analyzed using Stata version 13.0 and Graph Prism 6 software for the figures. The Mann-Whitney test for nonparametric variables was used to compare differences between individuals with high and low immune activation profiles. A *P* value of <.05 was considered statistically significant.

### Patient Consent Statement

All participants provided written informed consent for storage and future use of their samples in studies to understand host immune recovery during ART. The design of the work has been approved by the local ethical committee, the IDI research and ethics committee, and the Uganda National Council for Science and Technology, in Kampala.

## RESULTS

**Figure 3.**
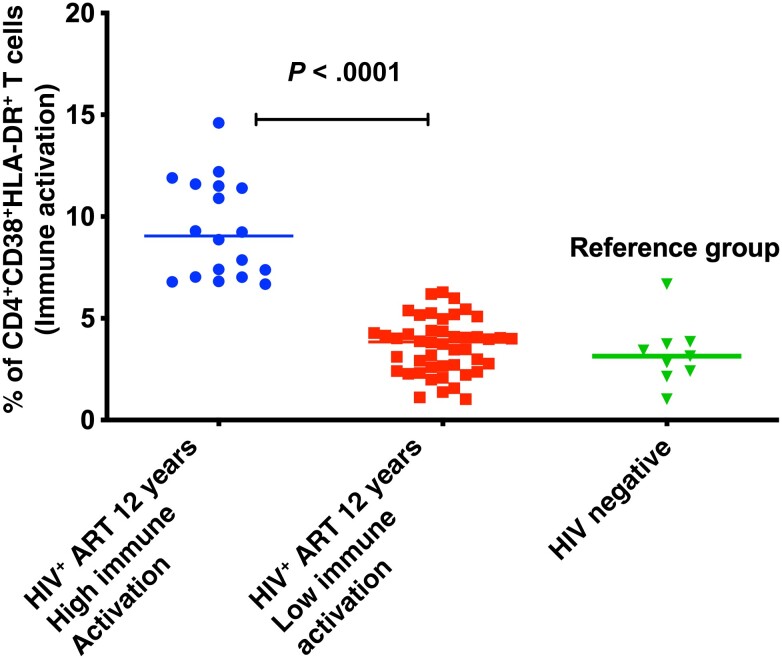
Immune activation phenotypes of long-term antiretroviral therapy–treated human immunodeficiency virus (HIV)–infected individuals after 12 years of treatment. Samples of HIV-negative individuals from the same community were used to provide background reference levels in the community. Abbreviations: ART, antiretroviral therapy; HIV^+^, human immunodeficiency virus positive.

Overall, 18 of the 58 ART-treated HIV-infected adults showed significantly high levels of immune activation (CD4/CD8^+^CD38^+^HLADR+ cells) in reference to the immune activation levels among age-and gender-matched HIV-negative persons from the same community (Figure 3). Among individuals with high immune activation, 11 (61%) were female and the median age was 50 (IQR, 42–61) years, with CD4 T cells ranging from 58 to 1018 cells/µL. In the low immune activation group, 28 (70%) participants were female and the median age was 49 (IQR, 39–65) years, with CD4 T cells ranging from 203 to 1587 cells/µL (Table 1).

**Table 1.** Demographic Characteristics of Human Immunodeficiency Virus–Infected Adults After 12 Years of Suppressive Antiretroviral Therapy

Characteristics	High Immune Activation (n = 18)	Low Immune Activation (n = 40)
Age, years, median (IQR)	50 (42–61)	49 (39–65)
Female sex, No. (%)	11 (61)	28 (70.0)
Baseline CD4 count, cells/μL, median (IQR)	97 (11–158)	97 (11–158)
Current CD4 count, cells/μL, median (IQR)	322.5 (58–1018)	814 (203–1587)
BMI, kg/m^2^, median (IQR)	21 (20–25)	25.95 (22–30)
cART duration, y, median (IQR)	13.1 (12.6–14.2)	13.1 (12.6–14.2)
Hypertension, No. (%)	2 (6.7)	2 (6.7)
Diabetes, No. (%)	2 (6.7)	1 (3.3)
Fever, No.	0	0
Current regimen, %		
ZDV/3TC/NVP	16	20
ZDV/3TC/EFV	16	22.5
TDF/3TC/EFV	33	17.5
TDF/3TC/DTG	35	40

All individuals started antiretroviral therapy (ART) at CD4 counts <350 cells/µL and had sustained viral suppression from the first viral load test after 6 months of ART.

Abbreviations: 3TC, lamivudine; BMI, body mass index; cART, combination antiretroviral therapy; DTG, dolutegravir; EFV, efavirenz; IQR, interquartile range; NVP, nevirapine; TDF, tenofovir disoproxil fumarate; ZDV, zidovudine.

### Caspase-1 Expression

**Figure 4.**
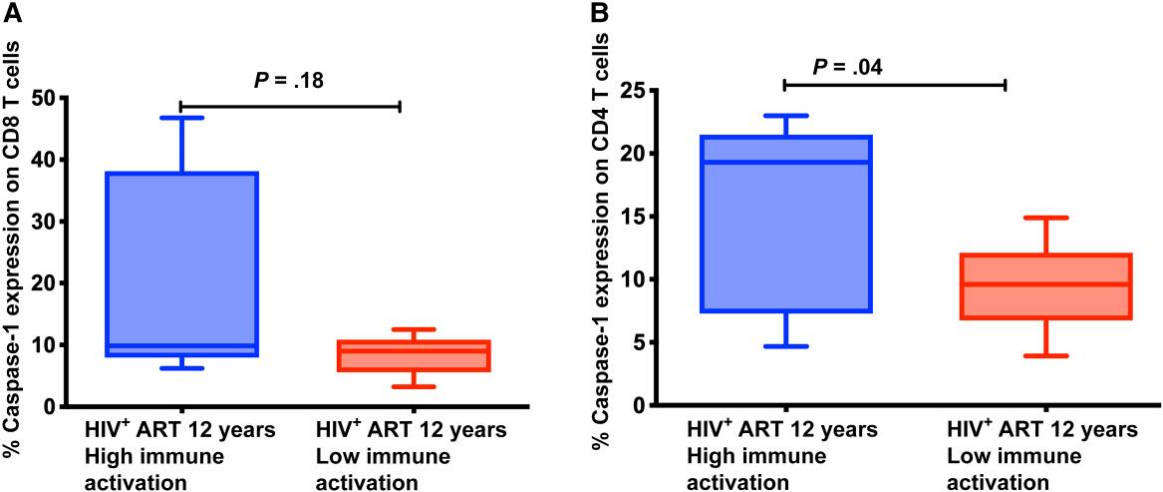
Expression of caspase-1 by CD4 and CD8 T cells among patients with persistent immune activation after 12 years of suppressive antiretroviral therapy. (*A*) shows percentage of CD8T-cells expressing caspase-1 and (*B*) shows percentatage of CD4 T-cells expressing caspase-1. The Mann-Whitney *U* test for nonparametric tests was used for comparisons between individuals with high and low immune activation (CD4^+^CD38^+^HLA-DR^+^ expression). Abbreviations: ART, antiretroviral therapy; HIV^+^, human immunodeficiency virus positive.

**Figure 5.**
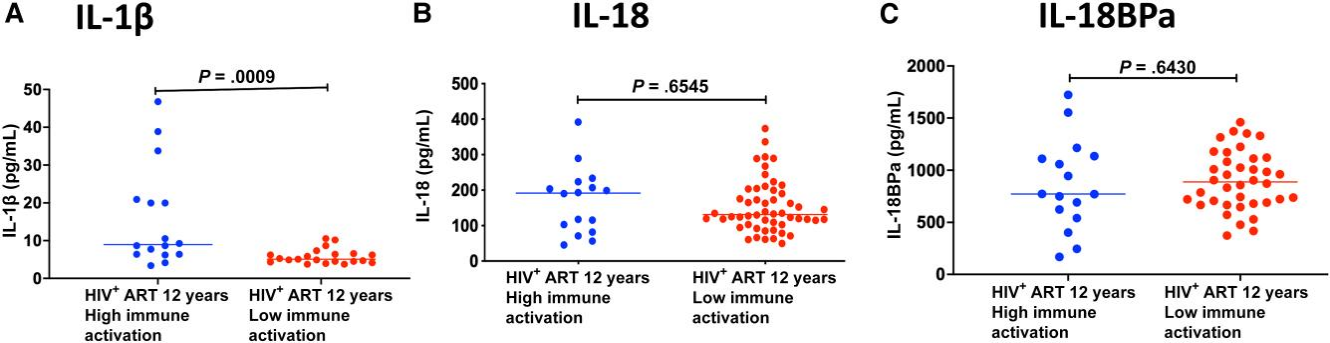
Levels of inflammasome byproducts in plasma. *A–C*, Levels of IL-1β (*A*), IL-18 (*B*), and IL-18BPa (*C*) among participants with high and low CD4 T-cell activation levels after 12 y of suppressive antiretroviral therapy. The Mann-Whitney *U* test for nonparametric tests was used for comparisons between high and low levels of immune activation (CD4^+^CD38^+^HLA-DR^+^ expression). Abbreviations: ART, antiretroviral therapy; HIV^+^, human immunodeficiency virus positive; IL, interleukin.

We observed higher percentages of caspase-1 expression from CD4 T cells in peripheral blood of individuals with higher immune activation levels (median, 19.30 [IQR, 7.285–21.5]) relative to individuals with lower immune activation (median, 9.6 [IQR, 6.74–12.10]) (*P* = .04; Figure 4). We further observed higher plasma levels of inflammatory byproduct IL-1β in individuals with higher immune activation levels (median, 8.945 [IQR, 6.398–20.69]) relative to individuals with lower immune activation (median, 5.077 [IQR, 4.416–6.492]; *P* = .009) (Figure 5). There was no observed difference in caspase-1 expression in CD8 T cells in peripheral blood of individuals with higher immune activation levels (median, 9.86 [IQR, 7.954–38.13]) relative to individuals with lower immune activation (median, 9.0 [IQR, 5.585–10.85]; *P* = .187) (Figure 4).

### Plasma Inflammasome by Products IL-18 and IL-18BPa

Plasma levels of inflammasome byproducts IL-18 and IL-18BPa were comparable among individuals with higher immune activation relative to individuals with lower immune activation (median, 191.7 [IQR, 87.14–219.4] and 131.4 [IQR, 104.6–219.4], *P* = .065; median, 771.9 [IQR, 561.9–1129] and 887.7 [IQR, 687.6–1114], *P* = .643, respectively) (Figure 5).

### Correlation of Immune Activation With Caspase-1 and Markers of Activated Inflammasome Pathway

**Figure 6.**
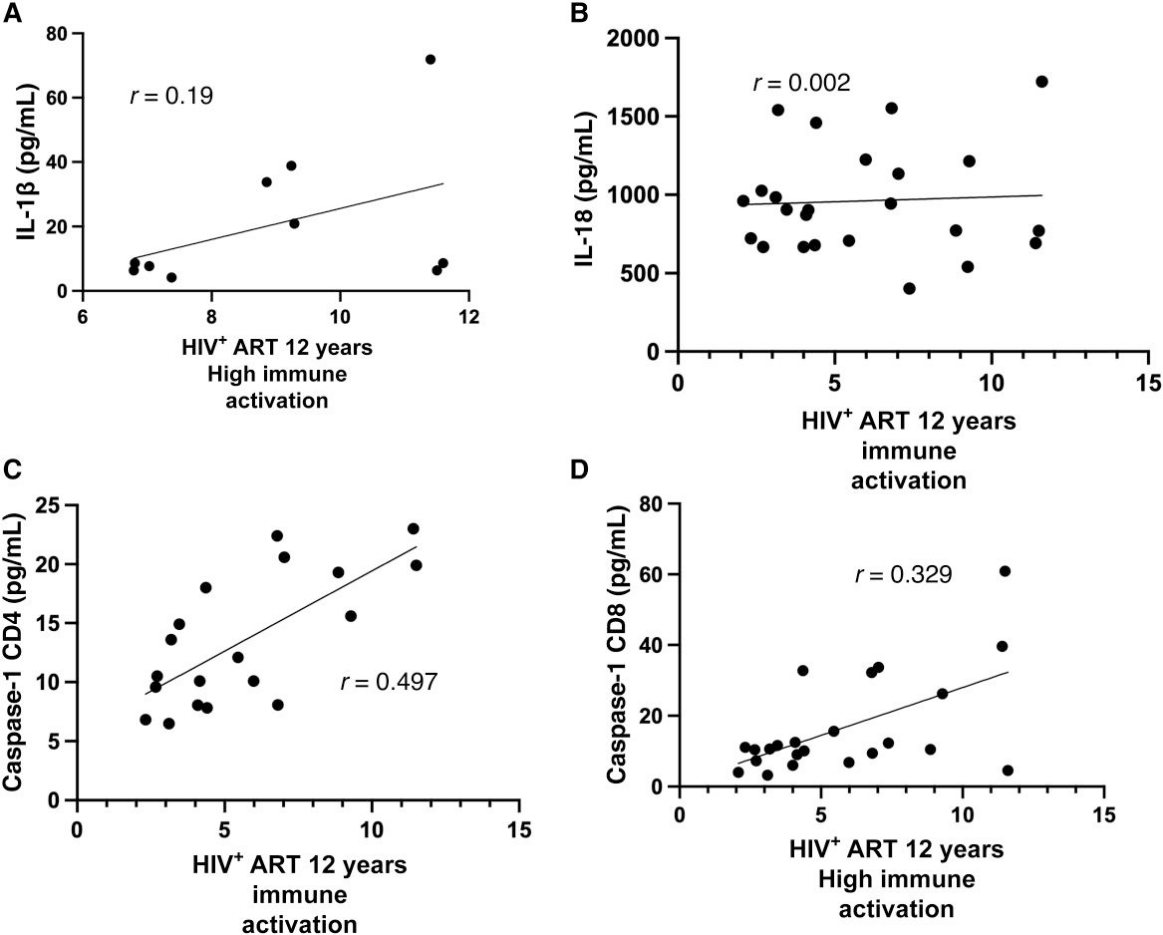
Correlation between immune activation and the inflammasome byproducts among antiretroviral therapy–treated human immunodeficiency virus–infected individuals after 12 y of treatment. *A–D*, Relationships between immune activation and interleukin 1β (*A*), immune activation and interleukin 18 (*B*), immune activation and caspase-1 expression by CD4 T cells (*C*), and immune activation and caspase-1 expression on CD8 T cells (*D*). Abbreviations: ART, antiretroviral therapy; HIV^+^, human immunodeficiency virus positive; IL, interleukin.

**Figure 7.**
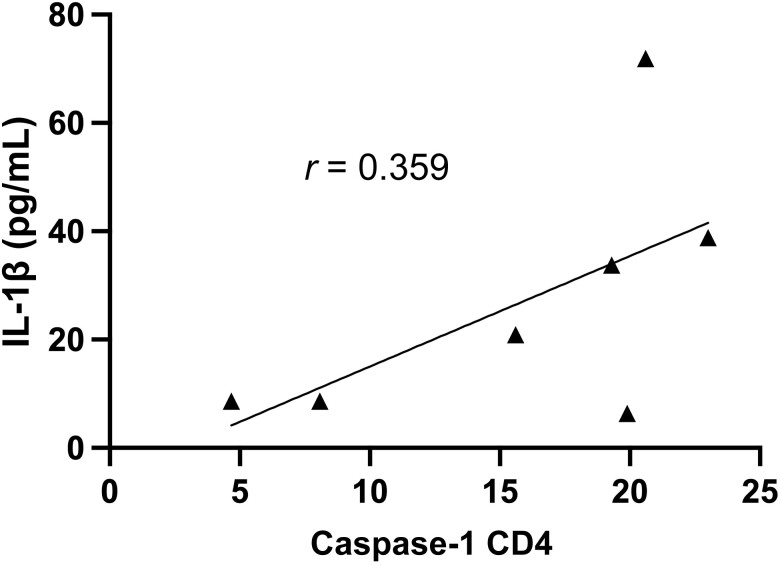
Correlation between interleukin 1β (IL-1β) and caspase-1 expression on CD4 T cells among antiretroviral therapy (ART)–treated human immunodeficiency virus–infected individuals with high immune activation after 12 y of suppressive ART.

We observed a low positive correlation between caspase-1 expression by both CD4 and CD8 T cells and immune activation levels (*r* = 0.497 and *r* = 0.329, respectively). IL-1β production showed a weak positive correlation with high immune activation levels (*r* = 0.19). There was no correlation observed between plasma levels of IL-18 and immune activation (Figure 6). We also observed that as caspase-1 expression on CD4 T cells increased, plasma levels of IL-1β increased (*r* = 0.359) (Figure 7).

## DISCUSSION

Antiretroviral therapy has improved livelihoods of HIV-infected individuals, but immune system abnormalities still exist, putting ART-treated individuals at risk of illnesses associated with immune activation and inflammation. We found evidence of activated pyroptosis, a highly inflammatory form of programmed cell death [16, 17] and the inflammasome pathway (caspase-1, IL-1β, and IL-18) among HIV-infected adults after 12 years of suppressive ART and persistent immune activation in the IDI HIV treatment cohort in Uganda. We report higher caspase-1 expression from CD4 T cells in peripheral blood of ART-treated individuals with high immune activation levels relative to individuals with low immune activation. Similarly, plasma levels of inflammasome byproduct IL-1β were higher in individuals with high immune activation levels relative to individuals with low immune activation. Pyroptosis can stimulate new rounds of pyroptosis, creating a feed-forward positive loop favoring chronic inflammation [23]. Although the mechanism is not yet well understood, it is plausible that ongoing production of low levels of HIV virus from the pool of latently infected cells in tissues might contribute to presence of short HIV transcripts in circulation that drive persistent immune activation and inflammation. Similarly, low production of virions from reactivated reservoir cells could result in abortive infection in presence of ART, leading to activation of inflammasome and proinflammatory caspases responsible for release of proinflammatory cytokines (IL-1β and IL-18).

Caspase-1 is a part of the family of intracellular cysteine proteases responsible for the maturation of pro-IL-1β and pro-IL-18, into mature forms. These are 2 related cytokines with critical roles in inflammation [28]. We anticipate that elevated levels of caspase-1 could be due to ongoing low levels of abortive infection among those ART-treated individuals with persistent immune activation despite peripheral HIV viral suppression. Abortive infection leads to impaired reverse transcription, hence presence of reverse transcripts, death of abortively infected cells by pyroptosis, and release of inflammasome byproducts such as caspase-1 [29].

The inflammasome byproducts contribute to chronic immune activation and inflammation observed among African ART-treated adults despite many years of ART [29]. Once pro-caspase-1 is activated to active caspase-1, there is cleavage of pro-IL-1β and pro-IL-18 into their mature, biologically active forms. IL-1β has been linked to many immune reactions, including the recruitment of inflammatory cells to the site of infection. IL-1β contributes to the control of bacterial, viral, parasitic, and fungal infections and has been associated with protection against tuberculosis [30]; however, its persistent production in the absence of infection has been associated with chronic immune activation and inflammation [15]. Immune activation and inflammation would be considered a positive response in the presence of an antigen but their persistence is linked to increased apoptosis, senescence, exhaustion, and anergy of immune cells in vivo [21, 22]. The increased levels of caspase-1 and IL-1β exhibited by ART-treated HIV-infected adults with HIV viral suppression (without coinfections) may contribute to noncommunicable diseases risk among ART-treated adults with chronic immune activation among adults aging with HIV [24–26]. Activation of IL-1β has been associated with a high inflammatory environment in some vital organs such as adipose tissue and pancreatic beta cells, resulting in metabolic disorders [31, 32]. A high inflammatory milieu among ART-treated HIV-infected individuals has been associated with accelerated occurrence of diseases of aging such as blinding cataracts that were associated with elevated peripheral cytokines [33] and occurred 2 decades earlier among HIV-infected (ART-treated) adults relative to their HIV-negative counterparts [34]. Although the exact mechanisms are still not yet well understood, it is clear that further research is needed to understand the mechanisms and drivers of chronic inflammation during long-term ART, which likely contributes to multisystem complications, particularly in Africa where other causes of inflammation are endemic.

On the contrary, we report similar levels of IL-18 in peripheral blood of ART-treated individuals with high and low immune activation levels. This was an inconsistency because, according to the inflammasome platform, activation of caspase-1 promotes the secretion of the proinflammatory cytokines IL-1β and IL-18 [15]. So, we expected IL-18 to be elevated among individuals with high caspase-1 and IL-1β. Previous work on gene expression, synthesis, and secretion of IL-18 and IL-1β show that although both IL-18 and IL-1β require processing by caspase-1, IL-1β is expressed only in presence of an abnormal immune environment, whereas IL-18 is constitutively expressed even in healthy individuals [35]. Therefore, this needs to be further studied because previous studies on the inflammasome pathways were not done in the setting of other endemic parasitic infections that are endemic in our African communities. It could also be explained by the fact that some immune repair pathways are only partially restored by ART, as shown in our previous studies that demonstrate persistent dysfunction of natural killer cells, monocytes, and innate lymphoid cells, despite restoration of CD4 counts to restoration of CD4 counts to relatively normal levels of peripheral CD4 counts of ≥500 cells/μL [36]. We also showed varied restoration of innate immune responses in ART-treated individuals receiving long-term ART [8, 9, 37, 38], which is also likely due to the fact that these individuals initiated ART after chronic HIV infection with severe immunosuppression of CD4 counts <350 cells/μL.

This study demonstrates a positive correlation between the inflammasome byproducts and immune activation. Although the correlation was quite low, it indicates an association between persistent immune activation and the inflammatory cell death pathways. One important limitation of this study is that these measurements were cross-sectional; we recommend longitudinal follow-up of these mechanistic pathways among ART-treated adults in SSA to better understand the causation and consequences of persistent immune activation and inflammation including understanding the role of inflammasome byproducts as well as Nef-1 protein, gasdermin-D, and latent reservoir characteristics on persistent immune activation and inflammation during long-term suppressive ART for treatment of chronic HIV infection in SSA. We did not test for helminths among these individuals, but this was unlikely to affect our results because acute clinical illnesses were excluded during routine clinical review of the research cohort participants before recruitment in our study; only individuals without any illness in the preceding 6 months were included. In addition, the reference group of HIV-negative persons from the same community with similar exposures controls for potential confounders. Our results underscore the complexity of managing chronic inflammation in HIV-infected individuals and the importance of considering multiple factors influencing immune activation and cell death pathways.

## CONCLUSIONS

High caspase-1 and IL-1β levels were observed among individuals with high immune activation despite 12 years of suppressive ART. There is a need to further understand the role of persistent abortive infection and the latent HIV reservoir characteristics as drivers of persistent activation and inflammation and to subsequently intervene to prevent the complications of chronic immune activation among adults receiving lifelong ART,

## Notes

***Author contributions.*** All authors contributed to the conceptualization and execution of the project and interpretation of the results. R. N. and D. N. contributed to the clinical and laboratory assays and drafted the manuscript. All authors reviewed and approved the manuscript for publication.

***Data availability.*** All data will be made available upon request.

***Financial support*.** This work was supported by the Fogarty International Center of the National Institutes of Health (award number D43TW009771). The authors also acknowledge funding from the European and Developing Countries Clinical Trials Partnership (EDCTP) (grant number TMACDF2020). The authors also acknowledge the DELTAS Africa (https://scienceforafrica.foundation/deltas-africa) (grant number 107743/Z/15/Z) that funded D. N. and R. N. through a group leader award.
